# Severity of depression, anxious distress and the risk of type 2 diabetes – a population-based cohort study in Sweden

**DOI:** 10.1186/s12889-019-7322-z

**Published:** 2019-08-27

**Authors:** Anna Deleskog, Rickard Ljung, Yvonne Forsell, Alicia Nevriana, Aysha Almas, Jette Möller

**Affiliations:** 1 Institute of Environmental Medicine, Unit of Epidemiology, Karolinska Institutet, SE 171 77, Stockholm, Sweden; 2 Department of Public Health Sciences, Karolinska Institutet, SE 171 77, Stockholm, Sweden; 3 Department of Medicine, Aga Khan University, Karachi, Pakistan

**Keywords:** Type 2 diabetes, Depression, Anxiety, Longitudinal data

## Abstract

**Background:**

Previous studies assessing the relationship between depression and diabetes mellitus did not consider the severity of depression. In the present study we assessed the risk of developing type 2 diabetes mellitus (T2DM) among people with various severity of depression.

**Methods:**

*This prospective longitudinal cohort study included 9,936 individuals residing in Stockholm County, Sweden who responded to the baseline questionnaire in 1998–2000. The participants were followed from 1 year after the baseline up to 2015 for the occurrence of* T2DM, using the National Patient Register, Swedish Prescribed Drug Registers, and Cause of Death Register. *Depression and anxious distress were assessed using psychiatric rating scales and defined according to the Diagnostic and Statistical Manual of Mental Disorders (DSM-5).*

**Results:**

Depression was associated with a statistically significant increased risk of T2DM after adjusting for potential confounders (OR 1.48, CI 1.10, 1.99). The strongest association was observed for severe depression (OR 1.72, CI 1.15, 2.59). Further, those with depression, regardless of severity, and with concurrent moderate/severe anxious distress had an increased risk of T2DM (OR 1.73, CI 1.13, 2.63) compared to those with neither depression nor anxious distress.

**Conclusions:**

*The study adds evidence that depression is associated with a higher risk for developing* T2DM*, and* the association is stronger among people with severe depression.

**Electronic supplementary material:**

The online version of this article (10.1186/s12889-019-7322-z) contains supplementary material, which is available to authorized users.

## Background

Depression is one of the leading cause of disability worldwide and it is a serious disorder linked to diminished quality of life, medical morbidity and mortality with a lifetime prevalence ranging from approximately 7.8 to 14.8% [1, 2]. However, the prevalence of depression has been reported to be higher and almost doubled among people with type 2 diabetes mellitus (T2DM) [3]. Additional to several well known risk factors for developing T2DM, such as obesity, physical inactivity, sedentary lifestyle, and high fat intake, it has also been suggested that depression plays a role for an increased risk of T2DM [4, 5]. This has previously been shown in meta-analyses [5–8]. Furthermore, findings from a meta-analysis of cross-sectional, population-based studies suggest a positive association between depressive symptoms and insulin resistance [9]. A potential pathophysiological mechanism linking depression to T2DM is that depression increases the activity of the hypothalamus pituitary adrenal axis (HPA) and the sympathetic system leading to increased cortisol and adrenaline/noradrenaline as well as pro-inflammatory cytokines [10, 11]. These stress hormones have various metabolic effects and that can result in insulin resistance and subsequent T2DM [6, 10, 12]. However, increased HPA axis activity might not be the only potential mechanism between depression and increased risk of T2DM. Several studies have found that the use of antidepressant medications are associated with increased risk of T2DM, possibly through its effect on weight gain [13, 14]. Despite many studies done to investigate the relationship between depression and T2DM, there is only a limited amount of longitudinal studies taking into account the severity of depression to the risk of T2DM. Moreover, no known study has considered depression with anxious distress, which is a recent specifier for depression in the Diagnostic and Statistical Manual of Mental Disorders (DSM-5), with the risk of T2DM. It has been known that depression with anxious symptoms is associated with poorer health outcomes [15].In this population-based cohort study, we aimed to assess the association between depression and anxious distress and the risk of T2DM and to assess the association of severity of depression and concomitant symptoms of anxious distress.

## Methods

### Participants

**Fig. 1:**
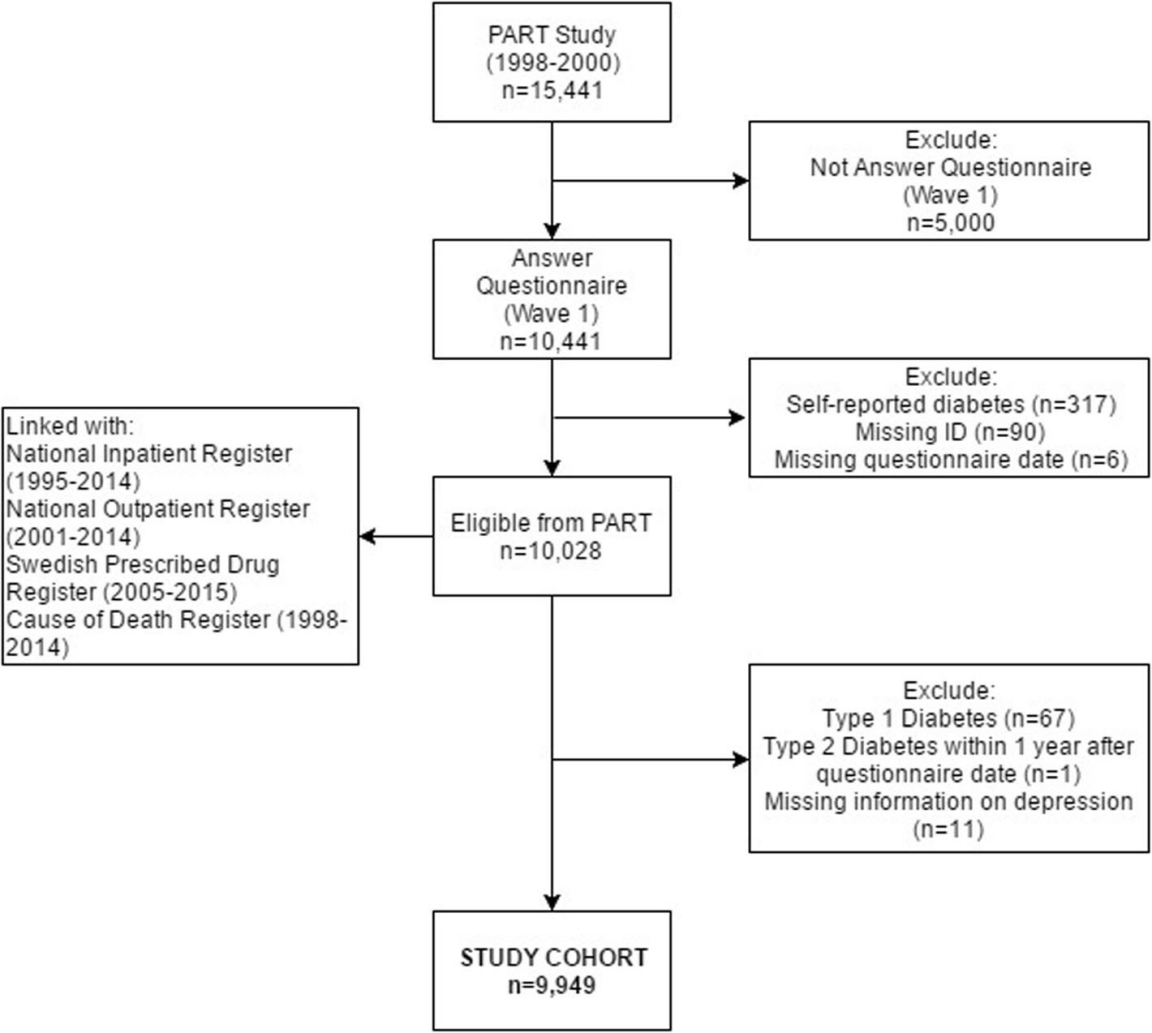
Study population

Data were derived from the PART study, a longitudinal cohort study of mental health (In Swedish short for Psykisk hälsa, Arbete och RelaTioner). The PART study included 10,441 individuals aged 20–64 years residing in Stockholm County, Sweden. Recruitment procedures, representativeness of the cohort and the study protocol have been described in detail elsewhere [16, 17, 18]. In brief, during the study period (1998–2015), postal questionnaires on depression and factors related to mental health were sent to the participants in three waves: wave 1 (1998–2000), wave 2 (2001–2003) and wave 3 (2010). Participants who reported diabetes in wave 1 or 2 and participants with missing ID and/or questionnaire date were excluded (*N* = 413) (Fig. 1). Data from the remaining participants were then linked to 83 the National Patient Register, Swedish Prescribed Drug 84 Register, and Cause of Death Register using the Swedish 85 personal identification number [19–21]. Using these registers, participants were followed from 1 year after the PART questionnaire assessment (in wave 1) until T2DM occurred, death, or 31 December 2015, whichever came first. We excluded participants with T1DM, participants diagnosed with T2DM in the immediate first year after the questionnaire date, participants with missing information on depression, and participants that died before the start of follow up (*N* = 92), leaving 9,936 participants as the final cohort. The average time between the start of follow-up to the first diagnosis of T2DM was 9.43 years. Figure 1 provides a detailed illustration of the participants’ selection. The study was approved by the Regional Ethical Review Board in Stockholm, Sweden (case number: 96–260, 01–218, 03–302, 2009/880–31, 2012/808–32). All participants gave their informed consent to participation.

### Exposure of depression and anxiety distress

In the PART study depression was evaluated using the Major Depression Inventory (MDI) and in the current study, as well as in previous publications [16, 17, 18], based on the responses given in wave 1 and wave 2. If a participant was depressed in both waves, the wave in which the MDI score was highest was used to determine the level severity. The MDI has shown high validity in clinical and non-clinical samples. [22]. The MDI scale consisted of 10 questions on symptoms present “nearly every day” during the previous 2 weeks. For each question there was five response alternatives scored from 1 to 5 according to the presence of the symptom: all the time (5), most of the time (4), slightly more than half of the time (3), slightly less than half of the time (2), some of the time (1) and never (0). The score on the 10 questions was summarized ranging from 0 to 50 [23]. Severity of depression was based on the MDI score and categorized as follows: not depressed (MDI score < 20), mild (20–24), moderate (25–29) and severe depression (≥30) [24]. The PART study was extensive and included multiple scales to assess mental health. Anxious distress was based on specific items corresponding to the DSM-5 from these scales and used as a specifier for depression. The moderate-severe category was combined with severe as the severe category requires a clinical observation (i.e. assessing motor agitation). It was defined as the presence of at least two of the following symptoms for the past 2 weeks: feeling keyed up or tense, feeling unusually restless, difficulty in concentrating because of worry, fear that something awful might happen and fear of losing self-control. Each symptom was assessed using a 5- or 6-point Likert scale, and, for simplicity, the response alternatives were merged as yes or no, and rated according to the number of DSM-5 symptoms. Anxious distress was then categorized based on the number of symptoms: no anxious distress (0–1), mild (2), and moderate/severe (≥3) [16, 17, 18].

### Outcome of T2DM

Incident cases of T2DM were assessed 1 year after responding to the questionnaire in wave 1. The first record of T2DM according to ICD-10 code diagnoses (E11) in the National Patient Register (data available from 1998 to 2014), inpatient care as well as specialized outpatient care was considered as an incident case. Additionally, participants with a prescription of antidiabetic drugs (ATC code A10 [25]) from 1 January 2006 onwards, without any record of T2DM in the patient register and no prescribed antidiabetic drugs July 1st to December 31st 2005 were also regarded as an incident case of T2DM. T2DM was also identified from the Cause of Death Register (data available from 1998 to 2014) using ICD-10 code (E11). Participants with recorded diabetes before the start of follow-up were excluded. Since follow up started 1 year after wave 1 questionnaire and the depression could either be measured from wave 1 or 2, we also assured that none of the participants was diagnosed between 1 year after wave 1 and wave 2. No participant was diagnosed with T2DM within this time period.

### Covariates

Age, sex and socioeconomic position were considered as potential confounders since they are associated with both depression and T2DM but does not come in the causal pathway between depression and diabetes. In addition, we considered smoking, alcohol use, physical activity and body mass index (BMI) as potential mediators, presenting different models taking these factors into account [26–31]. These factors have previously been shown to increase the risk of depression and T2DM [27–31]. While they might change as a consequence of depression, in this study, we decided to evaluate their influence as being in the pathways between depression and T2DM, as illustrated in Additional file 1: Figure S1. All these variables were measured through PART questionnaires.

Socioeconomic position was determined based on occupational status and main activity of the day in wave 1, as categorised by the Nordic Standard Occupational Classification (NSOC) of 1989 [32]. Socioeconomic positions were further grouped into the following: unskilled/semi-skilled workers, skilled workers, assistant non-manual workers, student, old-age or early retirement, self-employed, and high and intermediate level salaried employees.

Smoking habits were assessed in wave 2 and participants were asked whether they smoked regularly, smoked sometimes, had stopped smoking, or had never smoked. Hazardous alcohol use was identified using the Alcohol Use Disorders Identification Test (AUDIT) tool both in wave 1 and 2 [26]. The AUDIT tool consisted of 10 questions and the score ranges from 0 to 40 [26]. In this study, the results from AUDIT were dichotomized based on Swedish cut-off point (≥8 points for men and ≥ 6 for women) [24].

Information on physical activity was obtained in wave 2, through the following question, *“Do you exercise regularly, i.e. 2-3 times a week?”* in which the participants could answer *“yes”* or *“no”*. BMI (kg/m^2^) was calculated through self-reported weight and height in wave 1 and 2. It was further categorised into 4 groups: underweight (< 18.50), normal (18.50–24.99), overweight (25.00–29.99), and obese (≥30.00) [33]. For the variables where the information was available in both waves (i.e. AUDIT score and BMI), the highest value was chosen to be included in the analyses.

### Statistical analyses

Variables were presented as percentages or mean, (standard deviation (SD)). Missing value imputation was performed for MDI if there were 1 or 2 missing answers out of the 10 MDI questions; otherwise, the response was left as missing (*n* = 11 (0.11%) wave 1 and *n* = 16 (0.20%) wave 2). The missing answers were imputed using the mean value of the response in respective questions. A similar strategy was also applied for anxious distress, although imputation was only performed if there was only one missing answer out of the five questions. For other covariates (socioeconomic position, smoking, alcohol use, physical activity, BMI), the missing observations were grouped into a separate category (“missing”). Logistic regression models with and without adjustment for confounders were estimated for assessing the association between depression and T2DM expressed as odds ratios (OR) with 95% confidence intervals (CI). A stepwise approach was used to assess the association between depression and T2DM; model 1 the crude association, model 2;adjusted for age and sex, model 3 adjusted for age, sex, and socioeconomic position, and model 4 further adjustment for smoking status, alcohol use, physical activity, and BMI. Those without depression were considered as the reference group. When depression and anxious distress were assessed together as an exposure, those who had neither depression nor anxious distress served as the reference.

We also performed stratified analyses by age at enrolment, where we divided the category into < 50 years or ≥ 50 years. All analyses were performed using SAS 9.3.

**Table 1:** Characteristics of participants stratified by depression status

Variables	Categories	All	Depressed	Not depressed	*p*-values
		N (9936)	N (1425)	N (8511)	
		n (%)	n (%)	n (%)	
Age at baseline	Mean (SD)	41.1 (12.4)	39.1 (11.9)	41.4 (12.5)	<.0001
Age at baseline	< 50 years	6880 (69.2)	1070 (75.1)	5810 (68.3)	<.0001
	≥50 years	3056 (30.8)	355 (24.9)	2701 (31.7)	
T2DM at follow up^a^	No	9573 (96.4)	1364 (95.7)	8209 (96.5)	0.1726
	Yes	363 (3.7)	61 (4.3)	302 (3.6)	
Age at T2DM onset^b^	Mean (SD)	61.1 (9.8)	57.3 (9.6)	61.8 (9.6)	<.0001
Age at T2DM onset^b^	< 50 years	46 (12.7)	13 (21.3)	33 (10.9)	<.0001
	≥50 years	317 (87.3)	48 (78.7)	269 (89.1)	
Sex	Men	4395 (44.2)	434 (30.5)	3961 (46.5)	<.0001
	Women	5541 (55.8)	991 (69.5)	4550 (53.5)	
Socioeconomic position	Unskilled and semi-skilled workers	1148 (11.55)	227 (15.93)	921 (10.82)	<.0001
	Skilled workers	644 (6.48)	92 (6.46)	552 (6.49)	
	Assistant non-manual workers	1398 (14.07)	211 (14.81)	1187 (13.95)	
	Students	537 (5.40)	88 (6.18)	449 (5.28)	
	Retired	368 (3.70)	108 (7.58)	260 (3.05)	
	Self-employed (other than professional)	691 (6.95)	74 (5.19)	617 (7.25)	
	High and intermediate level salaried employee	4567 (45.96)	477 (33.47)	4090 (48.06)	
	Missing	583 (5.87)	148 (10.39)	435 (5.11)	
BMI (kg/m^2^)	Mean (SD)	24.2 (3.7)	24.1 (4.1)	24.3 (3.7)	0.1552
BMI	Normal (18.50–24.99)	6172 (62.1)	875 (61.4)	5297 (62.2)	0.0008
	Underweight (< 18.50)	202 (2.0)	44 (3.1)	158 (1.9)	
	Overweight (25.00–29.99)	2799 (28.2)	371 (26.0)	2428 (28.5)	
	Obese (≥30.00)	644 (6.5)	114 (8.0)	530 (6.2)	
	Missing	119 (1.2)	21 (1.5)	98 (1.2)	
Physical activity^c^	Yes	4416 (44.4)	533 (37.4)	3883 (45.6)	<.0001
	No	3780 (38.0)	661 (46.4)	3119 (36.7)	
	Missing	1740 (17.5)	231 (16.2)	1509 (17.7)	
Smoking	Regular smoker	1230 (12.4)	287 (20.1)	943 (11.1)	<.0001
	Occasional smoker	870 (8.8)	145 (10.2)	725 (8.5)	
	Ex-smoker	2380 (24.0)	310 (21.8)	2070 (24.3)	
	Never smoker	3686 (37.1)	452 (31.7)	3234 (38.0)	
	Missing	1770 (17.8)	231 (16.2)	1539 (18.1)	
Hazardous alcohol use^d^	Yes	2530 (25.5)	527 (37.0)	2003 (23.5)	<.0001
	No	7360 (74.1)	888 (62.3)	6472 (76.0)	
	Missing	46 (0.5)	10 (0.7)	36 (0.4)	
Severity of depression	No		8511 (85.7)		
	Mild		522 (5.3)		
	Moderate		345 (3.5)		
	Severe		558 (5.6)		
Anxious distress symptoms	No	7855 (79.1)	384 (27.0)	7471 (87.8)	<.0001
	Mild	1392 (14.0)	499 (35.0)	893 (10.5)	
	Moderate/severe	684 (6.9)	542 (38.0)	142 (1.7)	
	Missing	5 (0.1)	0 (0.0)	5 (0.1)	

Values expressed as mean and standard deviation (SD) for continues variables or number and percentage (%) of subjects in groups

*BMI* body mass index, *T2DM* Type 2 Diabetes Mellitus

^a^First record of T2DM using ICD-10 code (E11) in the National Patient Register more than one year after responding to the questionnaire, or anti diabetic drugs dispensation (ATC code A10) in the Swedish Prescribed Drug Registers from 1 January 2006, or T2DM recorded in the Cause of Death Register using ICD-10 code (E11)

^b^Only persons with T2DM

^c^Defined as exercise regularly 2–3 times a week

^d^Assessed using AUDIT (The Alcohol Use Disorders Identification Test) using cut-off points ≥ 8 for men and ≥ 6 for women (15, 16)

## Results

In total, 9,936 participants were followed from 1998 to 2015 (Fig. 1).

The prevalence of depression at baseline measured by MDI was 14.3% (*n* = 1425), and 5.3% (*n* = 522) had mild depression, 3.5% (*n* = 345) had moderate depression and 5.6% (*n* = 558) severe depression (Table 1). The average time between the start of follow up to the diagnosis of T2DM was 9.4 years (SD 3.04). Depression was more prevalent among women, and further individuals with depression were younger, had a higher prevalence of obesity, were more often smokers and less often reported to be physically active. At the end of the follow-up period, 4.3% of individuals with depression had developed T2DM compared with 3.6% among individuals without depression.

**Table 2:** Association between depression status, anxious distress, and type 2 diabetes

Variables	Categories	Model 1	Model 2	Model 3	Model 4
		OR (95% CI)	OR (95% CI)	OR (95% CI)	OR (95% CI)
Depression status	No	1	1	1	1
	Yes	1.22 (0.92–1.61)	1.62 (1.21–2.16)	1.48 (1.10–1.99)	1.34 (0.98–1.82)
Level of depression	No depression	1	1	1	1
	Mild depression	1.20 (0.77–1.86)	1.65 (1.05–2.60)	1.55 (0.98–2.44)	1.47 (0.91–2.36)
	Moderate depression	0.81 (0.43–1.54)	1.07 (0.56–2.05)	0.99 (0.52–1.90)	0.90 (0.46–1.77)
	Severe depression	1.49 (1.01–2.20)	1.92 (1.29–2.87)	1.72 (1.15–2.59)	1.49 (0.97–2.28)
Depression with/without levels anxious distress	No depression or anxious distress	1	1	1	1
	Depression without anxious distress	1.18 (0.71–1.98)	1.58 (0.93–2.67)	1.49 (0.88–2.53)	1.29 (0.75–2.23)
	Depression with mild anxious distress	1.02 (0.63–1.65)	1.32 (0.81–2.17)	1.22 (0.74–2.00)	1.13 (0.67–1.88)
	Depression with moderate/severe anxious distress	1.43 (0.95–2.13)	1.93 (1.28–2.93)	1.73 (1.13–2.63)	1.58 (1.02–2.44)

Logistic regression analyses with type 2 diabetes (T2DM) as the outcome variable. *CI* Confidence Interval, *OR* Odds Ratio, *MDI* Major Depression Inventory

Model 1 Crude

Model 2 Adjusted for age and sex

Model 3 Adjusted for age, sex, socioeconomic position

Model 4 Adjusted for age, sex, socioeconomic position, smoking, alcohol use, physical activity, body mass index

Depression was in the crude model associated with future risk of T2DM (OR 1.22, CI 0.92, 1.61) and after adjustment for potential confounders, the association was statistically significant (1.48, CI 1.10, 1.99) (Table 2, model 3). After further adjustment for smoking, alcohol use, physical activity and BMI an increased risk estimate remained although statistically non-significant (1.34, CI 0.98, 1.82) (Table 2, model 4). In addition, participants who had severe depression had a higher risk for T2DM (1.72, CI 1.15, 2.59) after adjustment for potential confounders (age, sex, socioeconomic position) (Table 2, model 3). Those with moderated depression did not display an increased risk for T2DM (Table 2). Further, after considering lifestyle factors the association remained but not statistically significant (1.49, CI 0.97, 2.28) (Table 2, model 4). Similarly, those with depression, regardless of severity, and with concurrent moderate or severe anxious distress had a statistically significant increased risk of T2DM (OR 1.73, CI 1.13, 2.63) (Table 2, model 3), which remained statistically significant after taking lifestyle factors into account (Table 2, model 4).

**Table 3:** Association between depression status, anxious distress, and type 2 diabetes, stratified by age at enrolment

	Model 1	Model 2	Model 3	Model 4
	OR (95% CI)	OR (95% CI)	OR (95% CI)	OR (95% CI)
Age < 50 years				
Depression status				
No	1	1	1	1
Yes	1.71 (1.16–2.51)	1.90 (1.29–2.80)	1.70 (1.14–2.55)	1.56 (1.03–2.38)
Level of depression				
No depression	1	1	1	1
Mild depression	1.44 (0.77–2.70)	1.61 (0.86–3.02)	1.49 (0.79–2.81)	1.57 (0.81–3.02)
Moderate depression	1.41 (0.65–3.06)	1.55 (0.71–3.38)	1.42 (0.65–3.11)	1.31 (0.58–2.95)
Severe depression	2.14 (1.28–3.60)	2.41 (1.43–4.07)	2.09 (1.21–3.60)	1.70 (0.96–3.00)
Depression with/without levels anxious distress				
No depression or anxious distress	1	1	1	1
Depression without anxious distress	1.69 (0.85–3.38)	1.92 (0.96–3.84)	1.79 (0.89–3.61)	1.57 (0.76–3.24)
Depression with mild anxious distress	1.51 (0.81–2.83)	1.64 (0.87–3.07)	1.48 (0.78–2.80)	1.40 (0.72–2.72)
Depression with moderate/severe anxious distress	1.89 (1.09–3.27)	2.14 (1.23–3.73)	1.86 (1.05–3.28)	1.71 (0.94–3.10)
Age ≥ 50 years				
Depression status				
No	1	1	1	1
Yes	1.05 (0.69–1.61)	1.18 (0.77–1.82)	1.04 (0.67–1.61)	0.97 (0.61–1.53)
Level of depression				
No depression	1	1	1	1
Mild depression	1.27 (0.67–2.40)	1.47 (0.77–2.80)	1.32 (0.69–2.52)	1.25 (0.63–2.46)
Moderate depression	0.47 (0.15–1.50)	0.52 (0.16–1.68)	0.48 (0.15–1.55)	0.45 (0.14–1.46)
Severe depression	1.24 (0.67–2.28)	1.37 (0.74–2.52)	1.15 (0.62–2.15)	1.07 (0.56–2.06)
Depression with/without levels anxious distress				
No depression or anxious distress	1	1	1	1
Depression without anxious distress	0.93 (0.43–2.03)	1.11 (0.51–2.44)	1.01 (0.46–2.24)	0.91 (0.40–2.06)
Depression with mild anxious distress	0.82 (0.38–1.78)	0.89 (0.41–1.94)	0.80 (0.36–1.75)	0.72 (0.32–1.63)
Depression with moderate/severe anxious distress	1.39 (0.75–2.56)	1.54 (0.83–2.85)	1.29 (0.69–2.42)	1.26 (0.66–2.44)

Logistic regression analyses with type 2 diabetes (T2DM) as the outcome variable. *CI*,Confidence Interval, *OR* Odds Ratio, *MDI* Major Depression Inventory

Model 1 Crude

Model 2 Adjusted for sex

Model 3 Adjusted for sex and socioeconomic position

Model 4 Adjusted for sex, socioeconomic position, smoking, alcohol use, physical activity, body mass index

Furthermore, since there was an effect of age, we stratified individuals by age. The results indicated that the association between depression and T2DM was only present in individuals below 50 years (1.70, CI 1.14, 2.55) and not among those 50 years and older (1.04, CI 0.67–1.61) (Table 3).

## Discussion

Taking into account age and gender differences, participants with depression have a higher risk of T2DM. The association was more robust among those with severe depression but only present among those 50 years and younger. Increased risks remain after further adjustment for sociodemographic factors. When also considering lifestyle factors, increased effect estimates remain. However, the confidence intervals show no statistical significance. This can be either due to decreased power or indicating that lifestyle factors partly explain some of the association between depression and T2DM. In addition, the risk of T2DM was highest among individuals with depression who also reported moderate/severe anxious distress.

Meta-analyses supported a relationship between depressed mood and an increased incidence of T2DM [6, 8]. A recent one of 33 studies demonstrated that depressed people have 32% increased risk for developing T2DM [5]. However, the meta-analysis included several studies where the time sequence of depressive symptoms and the incidence of diabetes was unclear [5]. Our study further supports these findings and for the sequence being depression increases the risk of T2DM.

There could be several reasons for the varied findings [34]. Participants with depression might visit their physician more often and may thus be more likely to be recognized as having T2DM. Other explanations could be that some of the studies did not distinguish between the diagnosis of depression and depressive symptoms. Consequently, the definition of participants with depression might have varied contributing to heterogeneity. Further, different questionnaires and different cut-off levels were used to define depression and the diagnosis of diabetes was often assessed only through self-report which may lead to misclassification of exposure and outcome.

Furthermore, a recent case-control study assessing the history of depression and the prediction of T2DM showed a threefold increased risk among individuals with lifetime depression before the onset of diabetes [35]. Limitations of that study are its study design with a retrospective evaluation of depression as well as a single assessment of current depression.

The most comprehensive meta-analysis including longitudinal studies (cohort and case-control design) investigating depression as a predictor for the development of T2DM included 23 observational studies and 424,557 participants with a mean follow-up of 8.3 years showed a pooled hazard ratio of 1.38, CI 1.23–1.55 [8]. It is worthy of note that the majority of the studies included measured depressive symptoms with self-reported questionnaires and various thresholds, and the diagnosis of diabetes was assessed only through self-report. Similarly, our study found the effects of the same magnitude. In contrast, our study was based on T2DM on clinical diagnoses in the hospital and prescribed anti-diabetic medications.

Interestingly, a prospective Swedish population-based study found a 2-fold increased risk of pre-diabetes in men with depression but no effect in women for 8–10 years follow-up [36]. Moreover, previous studies investigating the association between depression and diabetes had indicated an increased risk among persons of younger age [37]. It is hypothesised that persons exposed to psychological stressors are more prone to develop chronic diseases at a young age [37]. Since we had a relatively young cohort with only one third above the age of 50 years at baseline, age-stratified, and fully adjusted analyses suffered from limited statistical power. However, our analyses indicated a higher risk of T2DM among persons younger than 50 (Table 1). There are possible pathophysiological mechanisms that may explain the link between depression and development of T2DM. Depression is associated with HPA axis dysregulation causing high cortisol and catecholamine levels and metabolic changes as central adiposity leading to insulin resistance, and T2DM [38, 39]. Furthermore, depressive symptoms are also associated with increased inflammation, and inflammatory markers are known in the pathophysiology of T2DM [40].

The strength of this study is the longitudinal design, the use of a population-based sample and the use of the MDI, a validated scale for measuring depression [21]. In addition, using the national registers, including patient and drug registers, to follow up on the outcome ensured that new cases of T2DM were identified and no participants were lost to follow-up. Another strength is that we were able to adjust for sociodemographic and T2DM risk related lifestyle factors. An alternative analytic approach to adjust for confounding is propensity score matching. We performed that as a sensitivity analysis, based on a model of age, sex, SEP, BMI, smoking and alcohol. The result did not change, and gives further robustness to our study. However, our study also has its own limitations. We used prescription of antidiabetic drugs as a proxy for T2DM diagnosis. In Sweden, antidiabetic drugs are only prescribed to those with a clinical diagnosis of diabetes and not available over-the-counter. Guidelines recommend initiation of glucose-lowering medication added to lifestyle measures in newly diagnosed patients with T2DM [41]. Therefore, if some cases were treated solely with the advice of lifestyle changes, we would misclassify some individuals as having no T2DM. This misclassification would, however, be non-differential between depressed and non-depressed. In this material we did not have reliable information about treatments for depression, which could include both pharmacological treatments, cognitive behavioural therapy as well as physical activity. Depression has been linked to weight gain, partly because of changes in lifestyle but also due to pharmacological treatment with antidepressants. Thus, it might have resulted in over or underestimation of the associations. We also measured lifestyle-related factors at one point in time and using single measurements (e.g. for physical activity), which might have led to residual confounding. We also do not know if the severity of depression might have changed or if there have been repeated episode of depression during the follow up period for the study. Future studies should pay attention to this.

In all, the present study shows a longitudinal association between depression and the incidence of T2DM for subjects aged less than 50 years and adds evidence that depression is linked to the incidence of T2DM. In addition, the findings show that depression with concurrent moderate/severe anxious distress significantly further adds to the risk of T2DM.

## Conclusion

Our study suggests that people with depression, especially those with severe depression have a higher risk of T2DM. Our study highlights the importance of improving care in individuals with depressive disorders as well as anxious distress symptoms in order to prevent the potential development of T2DM.

## Additional file

Additional file 1: **Figure S1.** Direct Acyclic Graph to inform variable. A supplementary figure to visulize with a Direct Acyclic Graph (DAG) how variables have been selected and treated in the analyses. (JPG 50 kb)

## Data Availability

The datasets generated and/or analyzed during the current study are not publicly available due to confidentiality but data is accessible from the corresponding author on reasonable request.

## Abbreviations

HPA: Hypothalamus Pituitary Adrenal Axis
MDI: Major Depression Inventory
T2DM: Type 2 Diabetes Mellitus
