# Correction to: Severity of depression, anxious distress and the risk of type 2 diabetes – a population-based cohort study in Sweden

**DOI:** 10.1186/s12889-019-7587-2

**Published:** 2019-09-13

**Authors:** Anna Deleskog, Rickard Ljung, Yvonne Forsell, Alicia Nevriana, Aysha Almas, Jette Möller

**Affiliations:** 1 Institute of Environmental Medicine, Unit of Epidemiology, Karolinska Institutet, SE 171 77 Stockholm, Sweden; 2 Department of Public Health Sciences, Karolinska Institutet, SE 171 77 Stockholm, Sweden; 3 Department of Medicine, Aga Khan University, Karachi, Pakistan

**Correction to: BMC Public Health**

**https://doi.org/10.1186/s12889-019-7322-z**

It was highlighted that the original article [1] contained an error in the flow chart in Fig. 1. The study population states that the study cohort is *n* = 9949 but the correct number is *n* = 9936. This Correction article shows the correct Fig. 1.

**Fig. 1:**
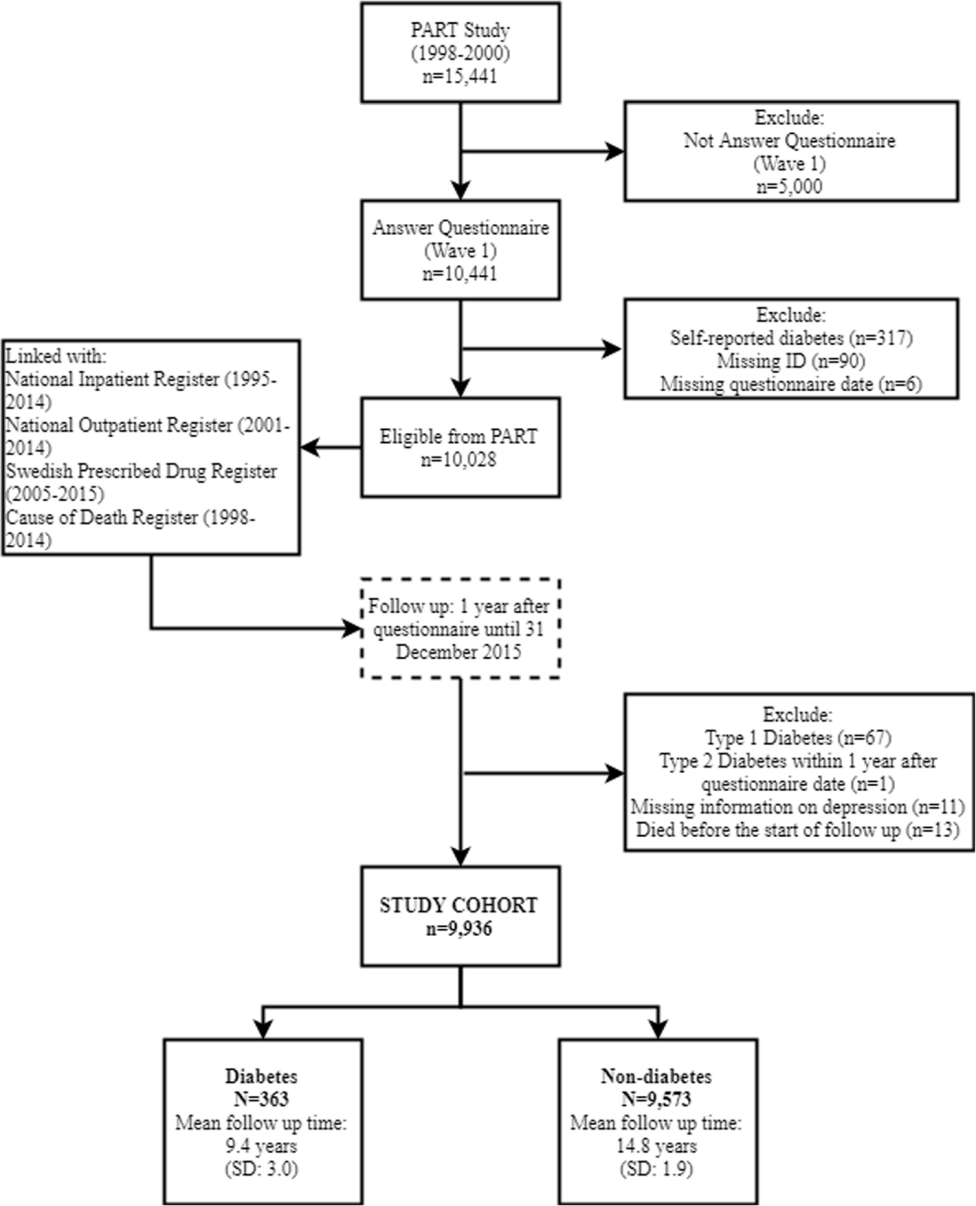
Study population
