# Supplementary figures and images for: Severity of depression, anxious distress and the risk of type 2 diabetes – a population-based cohort study in Sweden
Source: BMC Public Health. 2019 Aug 27;19:1174. doi: 10.1186/s12889-019-7322-z (PMC6712830; doi:10.1186/s12889-019-7322-z)

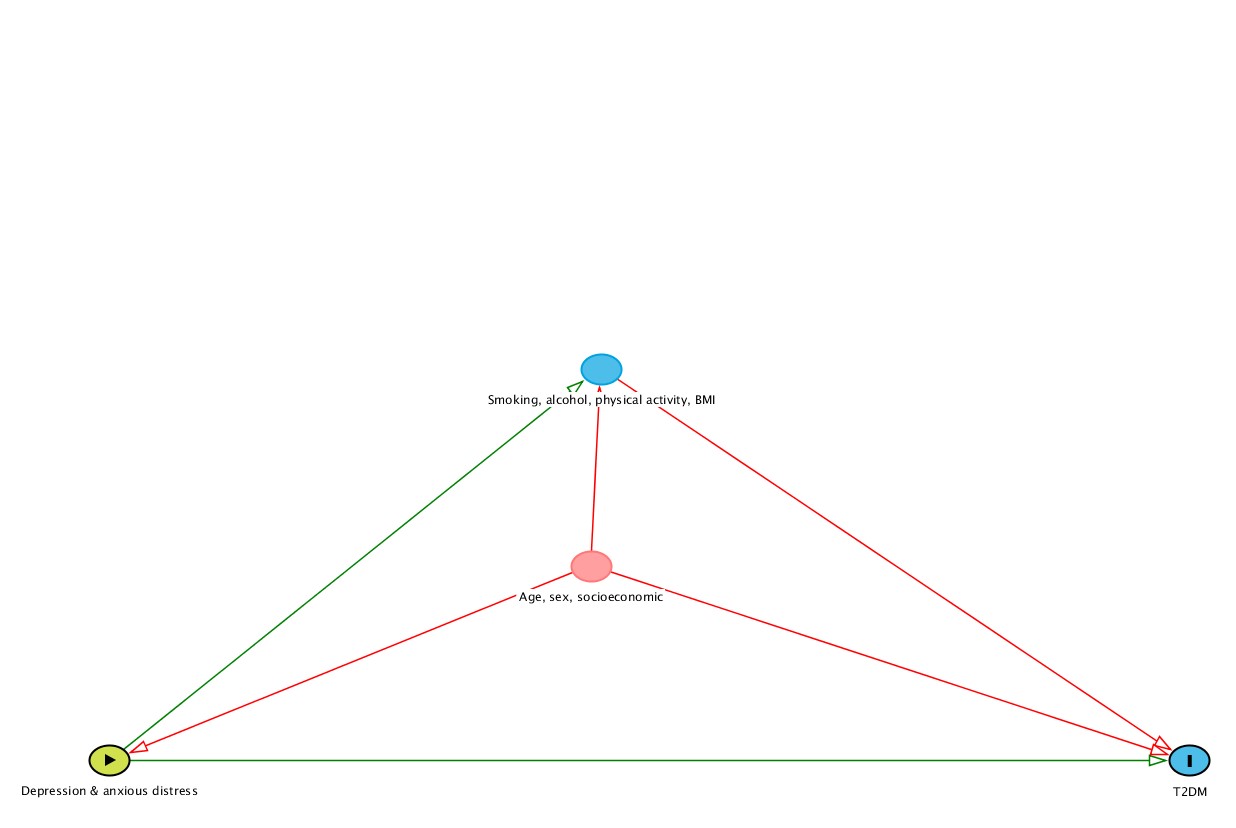

Supplement: Supplementary file 1 — Figure S1. Direct Acyclic Graph to inform variable. A supplementary figure to visulize with a Direct Acyclic Graph (DAG) how variables have been selected and treated in the analyses. (JPG 50 kb) [file 12889_2019_7322_MOESM1_ESM.jpg]
